# A fission yeast cell-based system for multidrug resistant HIV-1 proteases

**DOI:** 10.1186/s13578-016-0131-5

**Published:** 2017-01-11

**Authors:** Zsigmond Benko, Dong Liang, Ge Li, Robert T. Elder, Anindya Sarkar, Jun Takayama, Arun K. Ghosh, Richard Y. Zhao

**Affiliations:** 1 Department of Pathology, University of Maryland School of Medicine, Baltimore, MD 21201 USA; 2 Department of Microbiology-Immunology, University of Maryland School of Medicine, Baltimore, MD 21201 USA; 3 Institute of Human Virology, University of Maryland School of Medicine, Baltimore, MD 21201 USA; 4 Children’s Memorial Institute for Education and Research, Northwestern University Feinberg School of Medicine, Chicago, IL 10164 USA; 5 Department of Chemistry, Purdue University, West Lafayette, IN 47907 USA; 6 Department of Membrane Biochemistry, Institute of Animal Biochemistry and Genetics, Centre of Biosciences, SAS, 84005 Bratislava, Slovakia

**Keywords:** HIV-1, Multidrug resistant proteases, Fission yeast, Proteolytic cleavage, Cell proliferation, Oxidative stress, Mitochondria, Cell death, Protease inhibitors

## Abstract

**Background:**

HIV-1 protease (PR) is an essential enzyme for viral production. Thus, PR inhibitors (PIs) are the most effective class of anti-HIV drugs. However, the main challenge to the successful use of PI drugs in patient treatment is the emergence of multidrug resistant PRs (_mdr_PRs). This study aimed to develop a fission yeast cell-based system for rapid testing of new PIs that combat _mdr_PRs.

**Results:**

Three _mdr_PRs were isolated from HIV-infected patients that carried seven (_M7_PR), ten (_M10_PR) and eleven (_M11_PR) *PR* gene mutations, respectively. They were cloned and expressed in fission yeast under an inducible promoter to allow the measurement of PR-specific proteolysis and drug resistance. The results showed that all three _mdr_PRs maintained their abilities to proteolyze HIV viral substrates (MA↓CA and p6) and to confer drug resistance. Production of these proteins in the fission yeast caused cell growth inhibition, oxidative stress and altered mitochondrial morphologies that led to cell death. Five investigational PIs were used to test the utility of the established yeast system with an FDA-approved PI drug Darunavir (DRV) as control. All six compounds suppressed the wildtype PR (_wt_PR) and the _M7_PR-mediated activities. However, none of them were able to suppress the _M10_PR or the _M11_PR.

**Conclusions:**

The three clinically isolated _mdr_PRs maintained their viral proteolytic activities and drug resistance in the fission yeast. Furthermore, those viral _mdr_PR activities were coupled with the induction of growth inhibition and cell death, which could be used to test the PI activities. Indeed, the five investigational PIs and DRV suppressed the _wt_PR in fission yeast as they did in mammalian cells. Significantly, two of the high level _mdr_PRs (_M10_PR and _M11_PR) were resistant to all of the existing PI drugs including DRV. This observation underscores the importance of continued searching for new PIs against _mdr_PRs.

**Electronic supplementary material:**

The online version of this article (doi:10.1186/s13578-016-0131-5) contains supplementary material, which is available to authorized users.

## Background

HIV-1 Protease (PR) is an aspartic protease that normally presents as a homodimer with each subunit consisting of 99 amino acids (12kD) [1]. The active enzymatic site lies between two identical subunits and its activity can be blocked by the competitive binding of a specific PR inhibitor (PI) such as Indinavir (IDV) [2]. The primary function of HIV-1 PR is to proteolyze viral Gag-Pol polyprotein for the production of viral enzymes (reverse transcriptase, PR and integrase), structural proteins and the maturation of infectious viral particles [3–6]. Thus, HIV-1 PR is an essential enzyme for viral reproduction. Because of the important role of HIV-1 PR in HIV-1 infection, it is a major therapeutic target for antiretroviral therapies (ARTs). Indeed, HIV-1 PI is currently one of the most effective class of anti-HIV drugs. Monotherapy with PI alone can reduce HIV-1 viral loads by several logs [7]. When a PI drug is used in combination with other classes of anti-HIV drugs in treating HIV-infected patients, HIV-1 viral loads could be reduced to a level that often cannot be detected by the conventional laboratory methods [8, 9].

In spite of the tremendous progress we have made in ARTs, one of the main challenges to the success of ARTs is viral multidrug resistance (MDR) to the anti-HIV drug targets such as PR. The viral multidrug resistant PRs (_mdr_PRs) are developed primarily due to the continued emergence of viral gene mutations upon prolonged ARTs [10, 11]. Multidrug resistant HIV has been found in a significant portion of the adult populations in Africa that ranged from 10.6% to more than 50% [12]. HIV with MDR has also been found in pediatric patients globally [13]. Thus, constant effort is required to fight MDR.

One of the reasons why HIV-1 PRs are prone to the emergence of drug resistance is because all of the FDA-approved first generation PI drugs (_1st_PIs) are relatively weak inhibitors. They are small peptidomimetic drugs that were designed to mimic the natural HIV-1 PR substrates to compete bindings of the active enzymatic site [14]. Upon prolonged ART, the *PR* viral gene mutations could alter the configurations of the active enzymatic site that render the PIs non-effective in fitting into the active site. For example, a single I84V PR mutation could result in cross viral drug resistance to Fosamprenavir (FOS), Indinavir (IDV), Atazanavir (AZV), and Tipranavir (TPV) [15–17]. Consequently, none of the _1st_PIs are able to combat MDR. The second generation PIs (_2nd_PIs) are non-peptide P2 ligand-based small molecule drugs that were designed to combat MDR based on a new “backbone-binding” concept [17, 18]. This concept is based on the premise that if a P2 ligand-based PI binds to the S2 (or its symmetric counterpart S2’) subsite of the active enzyme with the maximum hydrogen-bonding affinity, it will prevent the PR from accessing its natural viral substrates thus inhibiting the HIV-1 PR activity. In addition, the tight P2-S2 binding affinity to the PR makes it more tolerable to viral *PR* gene mutations thus increasing the genetic barrier to the development of MDR [19, 20]. This new theory led to the development of the latest PI drug Darunavir (Prezista™, DRV) that was approved by FDA. Indeed, DRV inhibited _mdr_PRs even when most of the _1st_PI drugs failed. It also had a higher genetic barrier to the development of MDR than the other PI drugs [21].

In spite of these encouraging developments, MDR continues to occur including resistance to DRV [22–25]. For example, a clinical isolate that contains 20 multidrug resistant *PR* (_mdr_ *PR*) gene mutations was shown to exhibit extreme resistance to many of the PI drugs [22–24]. An in vitro selection study also showed that high levels of DRV resistance can be artificially selected in the laboratory [25]. These disturbing observations suggested that we need to continue developing new PIs that combat viral MDR.

Fission yeast (*Schizosaccharomyces pombe*) has been used as a model system in our laboratory to study the effects of HIV-1 viral protein R (Vpr) on cell proliferation, cell cycle G2/M regulation, and cell death/apoptosis over two decades [26–30]. A fission yeast cell-based high throughput screening system (HTS) for HIV-1 Vpr was developed [31]. This HTS platform was later adapted by the Molecular Libraries Probe Production Centers Network in the Molecular Libraries Program at NIH. Most recently, we showed for the first time that the wild type HIV-1 PR (_wt_PR) proteolyzes the HIV-1 viral substrates in fission yeast in the same manner as it does in the mammalian cells [32, 33]. As a follow-up of this initial finding, in this study, our primary goal was to develop a fission yeast cell-based system for functional analysis of HIV-1 _mdr_PRs. Our second objective was to validate and test some of the newer and _2nd_PIs in the established fission yeast system. Our long-term goal is to use the established fission yeast cell-based system for the development of high throughput screening system for new PI drug discovery and for testing of new PIs that are able to continue combatting viral MDR.

## Results

### HIV-1 _mdr_PRs cleave the same indigenous viral protein substrates in fission yeast

**Fig. 1:**
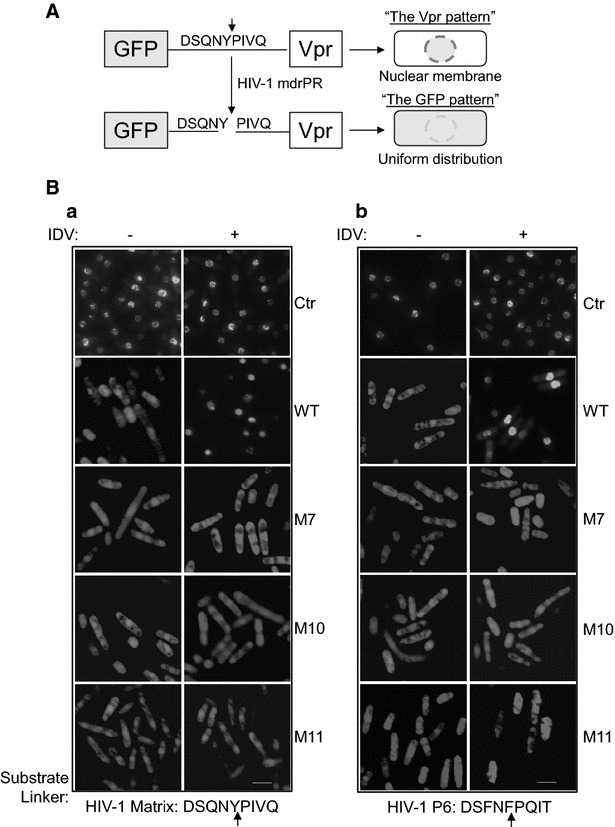
HIV-1 _mdr_PRs specifically cleave the GFP-MA-Vpr and the GFP-p6-Vpr fusion protein constructs that contain indigenous cleavage sites of HIV-1 p6 or MA viral proteins. **A** A schematic drawing to show how the proteolytic test was designed to measure the HIV-1 _mdr_PR-mediated cleavages of the GFP-MA-Vpr or the GFP-p6-Vpr fusion protein constructs in fission yeast. GFP, green fluorescent protein, which typically distributes uniformly throughout fission yeast cell, here we referred it as the “GFP pattern” [35, 52]. Vpr, HIV-1 viral protein R normally localizes predominantly on the nuclear membrane and appears as a “ring-like” structure. Thus we called it the “Vpr pattern” [35, 52]. The polypeptide shown was derived either from the HIV-1 MA↓CA (DSQNY↓PIVQ) or the p6 protein (DSFNF↓PQIT). The* arrow* indicates the PR cleavage site. **B** The GFP fluorescent images show the status of the HIV-1 _mdr_PRs-mediated cleavages against the GFP-MA-Vpr fusion protein construct (*a*) or the GFP-p6-Vpr construct (*b*) without (*left column*) or with the IDV treatment (*right column*). The cells were examined 20 h after the *PR* gene induction. *Arrows* indicate where the PR cleavage sites are. *Scale bar* 10 µm

Our prior results have shown that the _wt_PR cleaved the indigenous HIV-1 MA↓CA (DSQNY↓PIVQ) and p6 (DSFNF↓PQIT) viral targets in the fission yeast as it does in the HIV-1 infection of mammalian cells, suggesting that the protease activity of HIV-1 _wt_PR in fission yeast was similar to that in mammalian cells [32]. The experiment conducted here was aimed to test whether the three clinically isolated HIV-1 _mdr_PRs were also functional as PR enzymes in fission yeast. The same set of HIV-1 PR cleavage viral targets as described before for testing of the _wt_PR was used. Briefly, a “green fluorescent protein (GFP) re-localization assay” was used to specifically measure the proteolytic enzymatic activities of the HIV-1 PR in fission yeast. In this assay, two HIV-1 PR natural viral substrates MA↓CA and p6 were used to generate the “GFP-MA-Vpr” or the “GFP-p6-Vpr” gene fusion, in which each encodes a GFP for fluorescent detection, a MA↓CA (DSQNY↓PIVQ) or a p6 (DSFNF↓PQIT) substrate sequence [34], and a HIV-1 Vpr protein that is predominantly localized to the nuclear membrane [35]. Therefore, by design, without the PR, the production of these two fusion proteins in fission yeast will produce a “ring-like” structure on the nuclear membrane because of the property of Vpr, i.e., the “Vpr pattern” (Fig. 1A). Conversely, separation of GFP from Vpr due to the PR cleavage at either the MA↓CA (DSQNY↓PIVQ) or the p6 (DSFNF↓PQIT) substrate site will lead to the “GFP pattern,” i.e., with uniform distribution throughout cells [35]. Note that our early test results showed that the PR-mediated cleavages on the MA↓CA (DSQNY↓PIVQ) and on the p6 (DSFNF↓PQIT) substrates were target specific, and the _wt_PR by itself did not interfere with the intracellular localization of GFP or GFP-tagged Vpr in the fission yeast [32]. Therefore, we were able to measure the specific enzymatic activities of HIV-1 _mdr_PR as designed.

Indeed and as expected, both fusion protein constructs showed the “Vpr patterns” when no HIV-1 PRs were produced, suggesting the GFP-MA-Vpr or the GFP-p6-Vpr fusion protein was intact (Fig. 1B, upper rows). However, when either the *wtPR* or the _*mdr*_ *PR* were expressed in the fission yeast cells, the GFPs uniformly distributed throughout the cells that displayed the “GFP pattern”. The observed re-localization of GFP from the “Vpr pattern” to the “GFP pattern” suggested the HIV-1 PR-mediated cleavages resulted in separation of the Vpr from the GFP (Fig. 1B-a). Similar transitions from the “Vpr pattern” to the “GFP pattern” were also seen in the GFP-p6-Vpr fusion protein construct in both the _wt_PR- and the _mdr_PRs-producing cells (Fig. 1B-b).

To test whether HIV-1 _mdr_PRs retained their drug resistant status to some of the well-known PIs, an FDA approved PI, Indinavir (IDV, Crixivan™) was used to examine whether it could block the proteolytic HIV-1 _mdr_PR activities toward the GFP-MA-Vpr or the GFP-p6-Vpr construct (Fig. 1B). When IDV was added to the _wt_PR-producing cells, it prevented the _wt_PR protein cleavage activities [32]. This inhibitory activity was reflected by the reversion of the “GFP pattern” back to the “Vpr pattern”. In contrast, the same IDV treatments had no effects on the proteolytic activities of the _M7_PR, _M10_PR and _M11_PR as the cleavages of the fusion protein constructs were evident (Fig. 1B). These observations suggested these _mdr_PRs were still resistant to IDV. Together, these data showed that these three clinical _mdr_PR isolates maintained their viral proteolytic activities and drug resistance in the fission yeast.

### Production of HIV-1 _mdr_PRs prevent yeast colony formation, cell growth and lead to cell death

**Fig. 2:**
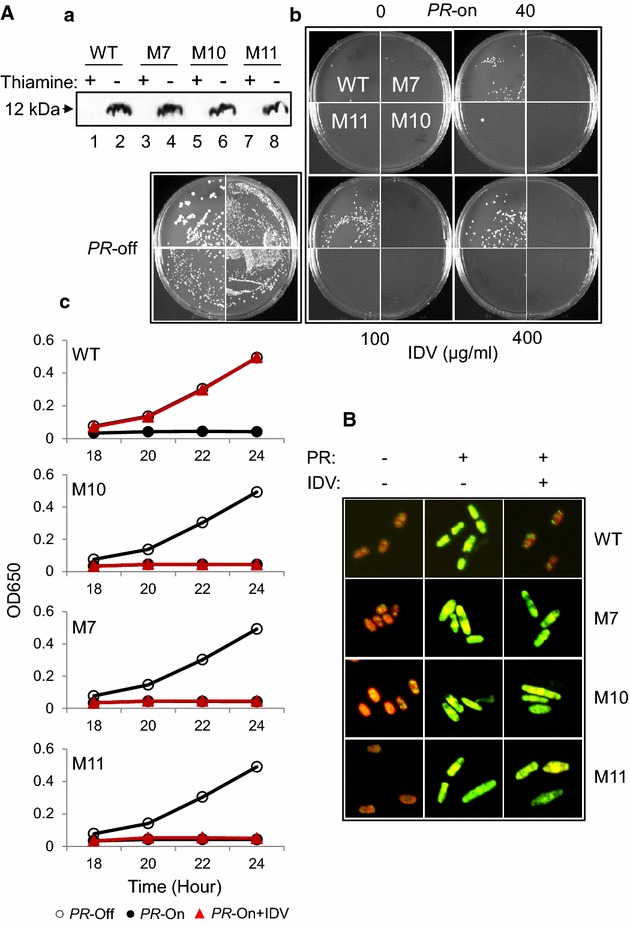
Production of HIV-1 _mdr_PRs prevent yeast colony formation, cell growth and lead to cell death. **A** The inducible expression of the _*wt*_ *PR* and _*mdr*_ *PR* genes in fission yeast 24 h after gene induction produced similar levels of HIV-1 PR proteins as detected by the western blot analysis (*a*), prevented the yeast colony formation (*b*) and the cellular growth over time (*c*). Note that the IDV treatment only prevented the effect of the _wt_PR but not the _mdr_PRs. The IDV concentration was added in (*b*) as shown. 100 µg/ml of IDV was added to the _*mdr*_ *PR*-expressing cells in (*c*). All cells were grown at 30 °C and the cell growth was measured by OD_650_ in the time period as indicated by using spectrophotometer. **B** Both the _wt_PR and _mdr_PR-induced cell death are shown by a yeast live/dead assay [27, 32]. Note that adding IDV before the gene induction only prevented the cell death induced by the _wt_PR (*upper row*) but not that by the _mdr_PR. Pictures were taken at 24 h after the gene induction. IDV (−), i.e., no IDV added; IDV (+), 100 µg/ml of IDV was added prior to the gene inductions

In our earlier report, we observed that HIV-1 _*wt*_ *PR* gene overexpression prevented yeast colony formation and cell proliferation leading to cell death [32, 33]. Here we would like to test whether HIV-1 _mdr_PRs would have the same effects on the fission yeast as the _wt_PR. A yeast colony formation assay was first used to test potential effects of the HIV-1 _mdr_PRs on cellular growth on agar plate. Prior to the test, the production of HIV-1 _mdr_PR proteins in each respective strain was first verified by the western blot analysis. As shown in Fig. 2A-a, a single band of 12 kDa protein was detected reacting to the anti-PR monoclonal antibody in each one of the pYZ1N-*PR* carrying fission yeast strains when they were under the gene-inducing (PR-on) condition without thiamine. In contrast, no PR protein band was seen when thiamine was added to suppress the *PR* gene expressions in the same cells (PR-off), indicating the specific PR protein production under the inducible *nmt1* promoter. Also note that the PR protein levels were very similar among all four PR-producing cells. The yeast colony formation was first measured in the *PR*-suppressive (*PR*-off) medium (Fig. 2A-b). Normal size colonies were seen in both of the _wt_PR and _mdr_PR cells (top). In contrast, when the same amounts of cells were plated on the *PR*-inducing (*PR*-on) agar plates, little or no colonies were observed (bottom).

To test whether the _mdr_PRs retained their status of drug resistance while they conferred the observed inhibitory effects on cellular growth and colony formation, IDV was added. As expected, when IDV was added to the agar plates producing the _mdr_PR at increasing concentrations between 40 and 400 µg/mL, colony formations were restored only on the _wt_PR-producing plate (Fig. 2A-b, left top). However, it had no reversing effects on any of the _mdr_PR-producing plates under all three different IDV concentrations, suggesting that these three _mdr_PRs remained resistant to IDV.

To have a more comprehensive understanding toward the effects of HIV-1 _mdr_PRs on cellular proliferation, the fission yeast cellular growth kinetics was measured. Same as the _wt_PR [32, 33], the expression of all three _mdr_PRs individually completely blocked cellular growth (Fig. 2A-c, closed circles). Conversely, the same cells under the *PR*-suppressing (*PR*-off) conditions grew normally with nearly indistinguishable growth kinetics (Fig. 2A-c, open circles). However, when IDV was added to the PR-producing medium prior to the gene induction, it completely reversed the effect of HIV-1 _wt_PR on the cellular growth but it had no effect on the _mdr_PRs (Fig. 2A-c, triangles).

We next tested whether, like the _wt_PR, prolonged HIV-1 _*mdr*_ *PR* expression kills fission yeast cells. A commercial yeast live/dead viability assay was used to determine the status of intracellular metabolism [32]. As shown in Fig. 2B, when no _mdr_PRs were produced in the fission yeast, cells showed an orange-red color suggesting viable and actively respiring cells (Fig. 2B, left column). However, 24 h after the HIV-1 _*mdr*_ *PR* genes were induced, the fission yeast cells turned to the greenish yellow (Fig. 2B, middle column) indicating that those cells were metabolically dead. Same as the inhibitory effect of IDV on PR-induced growth arrest, when IDV was added to the PR-producing medium prior to the gene induction, it completely prevented the _wt_PR-induced cell death as those cells remained orange-red. In contrast, the same treatment of IDV to the _mdr_PR cells had no effects on the _mdr_PRs-induced cell death because all of those cells turned into the same greenish yellow colors as the dead cells (Fig. 2B, right column). These observations suggested that the production of HIV-1 _mdr_PRs in the fission yeast prevented the colony formation and cell proliferation that led to cell death.

### Expression of HIV-1 _*mdr*_*PRs* induce cellular oxidative stress and changes in mitochondrial morphology

**Fig. 3:**
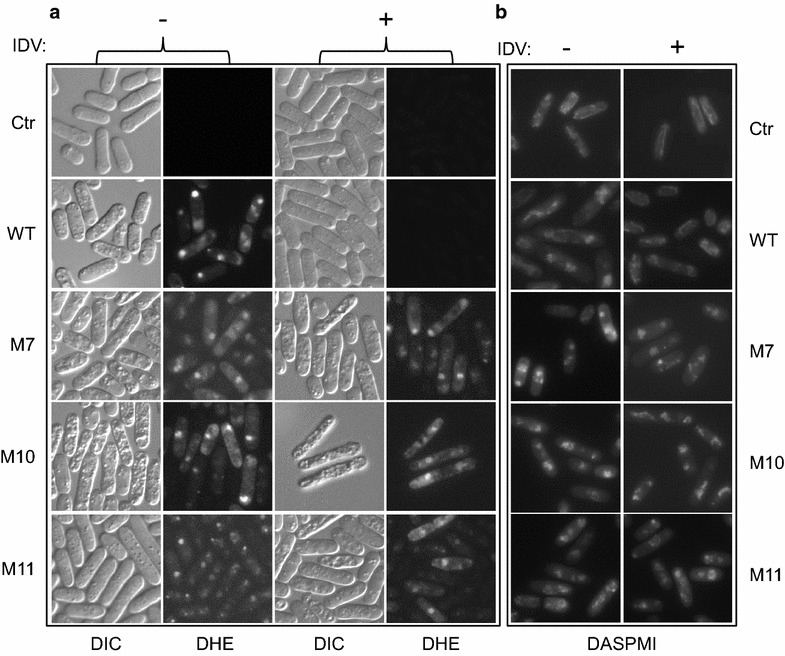
HIV-1 _mdr_PR induce oxidative stress and alteration of mitochondrial morphologies. Both of the _wt_PR and _mdr_PRs induced oxidative stress **a** and mitochondrial morphological changes **b** in fission yeast. Unlike the _wt_PR, the effects induced by _mdr_PRs were resistant to IDV. Production of reactive oxygen species (ROS) was measured by a ROS indicator dye DHE (**a**). The induction of ROS was shown by increased and visible staining of DHE. The mitochondrial morphologies (**b**) were visualized by staining of the fission yeast cells with a mitochondria-specific fluorescent probe DASPMI [29, 41]. Note that normal mitochondria typically appear like threads or necklaces constituted with multiple small dots concentrated around the edge, or as a tubular network extended along the cell peripheries. Changes in the mitochondrial morphologies were shown here as different sizes of mitochondrial aggregates that were situated almost randomly throughout _*mdr*_ *PR*-expressing cells. 100 µg/ml of IDV was added before gene induction of all *PR*-expressing cells. All cells were imaged 24 h after the gene induction

To explore the molecular mechanism underlying HIV-1 _mdr_PR-induced cell death, possible intracellular stress induced by HIV-1 _mdr_PRs was determined by the production of oxidative stress species (ROS) [29]. A ROS-specific dye, dihydroethidium (DHE), which produces red fluorescence in the presence of ROS, was used to measure cellular oxidative stress in the _*mdr*_ *PR*-expressing cells. As shown in Fig. 3a (left two columns), 24 h after the _*mdr*_ *PR* gene induction, strong red fluorescence was detected in both of the _*wt*_ *PR*- and the _*mdr*_ *PR*-expressing cells; whereas no red fluorescence was observed in the control cells (Fig. 3a, top row), suggesting that HIV-1 _mdr_PRs indeed induced the ROS production in the fission yeast. Consistent with the notion that the PR-induced ROS production was due to the PR enzymatic activities, the IDV treatment prior to the *PR* gene inductions prevented the production of ROS in the _wt_PR but not in the _mdr_PRs (Fig. 3a, right two columns).

Since HIV-1 _wt_PR also induces alteration of mitochondrial morphologies in fission yeast cells [32], we tested whether the _mdr_PRs have any effects on the mitochondrial morphology of the fission yeast cells. Morphology of fission yeast mitochondria was visualized by staining with a mitochondria-specific dye, 2-(4-dimethylaminostyryl)-1-methylpyridinium iodide (DASPMI) [29, 36]. Consistent with previous descriptions of normal fission yeast mitochondrial morphologies [32], the mitochondria in the control cells appeared as tubular networks extending along the periphery of the cells (Fig. 3b, top row). In contrast, the mitochondria aggregated in both of the _*wt*_ *PR* and the _*mdr*_ *PR*-expressing cells (Fig. 3b, left column), indicating abnormal mitochondrial morphologies. Consistent with the drug resistant status of the testing _mdr_PRs, the IDV treatment reversed the effect of the _wt_PR but not the effects of the _mdr_PRs (Fig. 3b, right column). Therefore, similar to the _wt_PR, the _mdr_PRs also caused changes in mitochondrial morphology in fission yeast cells.

### The second generation protease inhibitors darunavir and its derivatives suppress the _M7_PR but not the _M10_PR or _M11_PR

One of the ultimate goals of this study was to develop a fission yeast cell-based system that would allow us to test new PIs against those HIV-1 proteases that are already resistant to the existing protease inhibitory drugs. By developing different fission yeast strains that contain PRs with various levels of drug resistance, we were hoping to provide a platform to test the drug resistance of HIV-1 PR activities at low (_M7_PR) and high (_M10_PR or _M11_PR) levels of MDR.

**Fig. 4:**
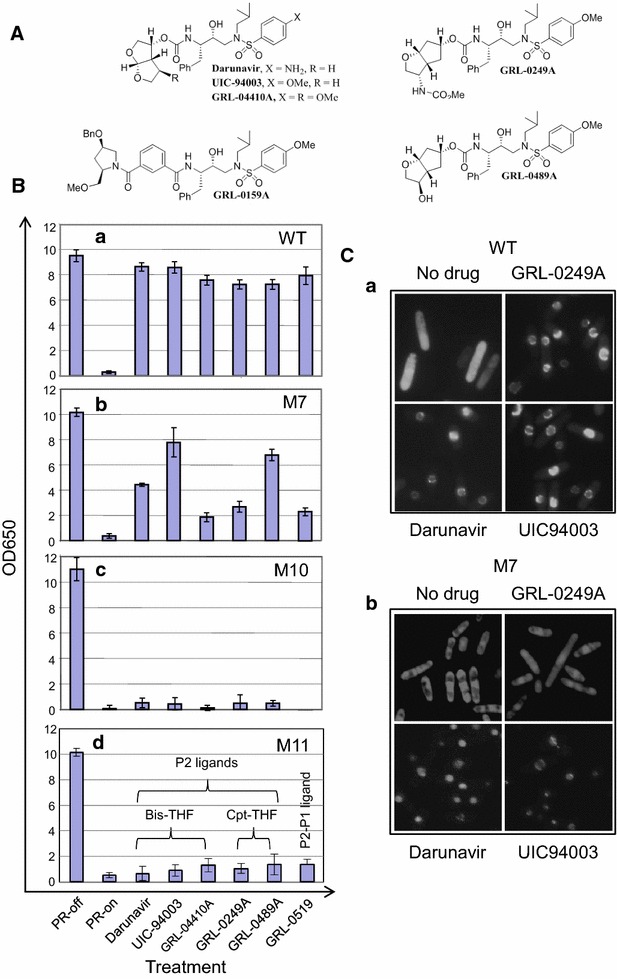
DRV and its derivatives suppress the _M7_PR but not the _M10_PR or _M11_PR. The chemical structures of protease inhibitors, DRV, UIC-94003, GRL-0489A, GRL-0159A , GRL-0249A and GRL-044-10A are shown in (**A**). All six compounds including DRV are P2 ligands [17]. Effects of the newly synthesized protease inhibitors on _mdr_PR-induced growth arrest were measured against the _wt_PR and _mdr_PRs by using a liquid growth assay and measured by OD_650_ at 48 h (**B**). The final drug concentration of 200 µM was used in each of the experiments. DRV was used here as a positive control and no drug treatment was used as a negative control. **C** Effects of the newly synthesize protease inhibitors on _mdr_PR-mediated protein cleavages were tested by using the GFP-p6-Vpr fusion protein construct as described in Fig. 1A. Only the effects of UIC94003 and GRL-0489A on the _wt_PR and the _M7_PR were tested here because of the initial results shown in (**B**). DRV was used here as a positive control and no drug treatment was used as a negative control, respectively. The _mdr_PR enzymatic activities were visualized under fluorescent microscopy 20 h after the gene inductions. THF, tetrahydrofuran; Cpt, cyclopentyl

To test this possibility, we tested six _2nd_PIs that included an FDA-approved drug, DRV and five research grade protease inhibiting compounds UIC-94003, GRL-044-10A, GRL-0249A, GRL-0489A and GRL-0159A (Fig. 4A). They are all P2 ligand-based PI compounds. The five research grade PIs are structurally related to DRV. The design and synthesis of those compounds were part of the effort to improve the P2-S2 bindings in order to escalate the genetic barrier to the development of MDR [17]. The UIC-94003 and GRL-044-10A are in the same structural class as the DRV; the GRL-0249A and GRL-0489A are in the group of cyclopentyl-THF (cpt-THF); and the GRL-0159A is unique [17]. Some of these inhibitors have been tested previously in mammalian cells and showed various degrees of inhibitory activities against HIV-1 _mdr_PRs [17, 37, 38].

To test whether these compounds had the same inhibitory effects in fission yeast as they did in mammalian cells, both of the _wt_PR- and _mdr_PR-producing yeast cells were treated with 200 µM of each compound prior to gene induction. The effects of these newly synthesized PIs on HIV-1 PR-induced growth arrest were measured against the _wt_PR and three _mdr_PRs by using a liquid growth assay. The yeast growth was measured by OD_650_ at 48 h (Fig. 4B) or over a time period of 16–72 h after the drug treatments (Additional file 1: Figure S1). No drug treatment and the DRV treatment were used here as the negative and positive controls, respectively. Consistent with the testing results in mammalian cells, all six compounds restored cellular growth in the _wt_PR-producing cells (Fig. 4B-a). Interestingly, however, different levels of suppression were observed among those compounds in the _M7_PR-producing cells (Fig. 4B-b). While DRV partially diminished the _M7_PR-induced growth arrest, the UIC-94003 showed the strongest suppression against the _M7_PR with nearly 80% recovery of the cellular growth and the GRL-0489A showed the second strongest suppression against the _M7_PR with about 60% recovery of the cell growth. However, none of the six compounds had any clear suppression activities against the _M10_PR or the _M11_PR (Fig. 4B-c-d; Table 1). Longitudinal testing of these 6 compounds against the _mdr_PR-induced growth arrest over time (Additional file 1: Figure S1) revealed essentially the same information as shown in Fig. 4B.

**Table 1:** Mutational and multidrug resistant profiles of proteases isolated from HIV-infected patients

Mutation status	Nonsynonymous gene mutations found in the *PR* gene	Known resistance to PI drugs	Level of drug resistance to protease inhibitors
WT	None	None	None
M7	V32I, L33I, M36I, I54 V, A71 V, G73S, L90 M	**IDV, SQV, RTV, NFV, ATV** (APV, FOS, LPV, TPV)	Low
M10	L10I, I13 V, K20R, L33I, M36I, I54 M, A71T, G73S, I84 V, L90 M	**APV, FOS, IDV, SQV, RTV, NFV, ATV, TPV** (LPV)	High
M11	L10F, L33F, M46I, I54L, H69 K, A71 V, G73S, V77I, V82T, I84 V, L90 M	**APV, FOS, IDV, SQV, RTV, NFV, ATV, TPV** (LPV)	High

The three mutant HIV-1 PRs were isolated from the plasma samples of HIV-infected patients who were cared at the University of Maryland Medical Center. They carried seven (M7), ten (M10) and eleven (M11) *PR* gene mutations, respectively. The wildtype (WT) PR was derived from pNL4-3. The drug resistant profiles were generated in a CAP/CLIA accredited hospital laboratory as part of the clinical reports by using the ViroSeq HIV-1 Genotyping System (Abbott Molecular, Chicago, IL). APV, Amprenavir; FOS, Fosamprenavir; IDV, Indinavir; SQV, Saquinavir; LPV, Lopinavir + Ritonavir; RTV, Ritonavir; NFV, Nelfinavir; ATV, Atazanavir; TPV, Tipranavir; Drugs in parenthesis indicate possible drug resistance

Since the compound UIC-94003 showed the strongest suppression against the _M7_PR-mediated growth arrest, we decided to test whether this compound could also block the proteolytic activities of the _wt_PR or the _M7_PR. We also picked GRL-0249A for the comparison purpose because it showed little or no suppression against the _M7_PR. In addition, the DRV treatment was used as a positive control and no drug treatment was used as a negative control, respectively. The effect of these two compounds on HIV-1 PR-mediated protein cleavages was tested by using the GFP-p6-Vpr fusion protein construct as described in Fig. 1A. The PR enzymatic activities were visualized by monitoring the cellular distribution of the GFP under fluorescent microscopy 20 h after the gene induction. As shown in Fig. 4C-a, the control _wt_PR-producing cells cleaved the GFP-p6-Vpr fusion protein construct as expected and showed the “GFP pattern” (Fig. 4C-a, left top). The treatment of the same _wt_PR-producing cells with any one of the three compounds completely blocked the _wt_PR activities resulting in the “Vpr pattern”. In contrast, only UIC94003 and DRV were able to block the _M7_PR-mediated protein cleavages (Fig. 4C-b, lower); whereas the GRL-0249A showed no sign of suppression against the _M7_PR-mediated cleavage (Fig. 4C-b, upper). Therefore, these testing results shown here suggested that, consistent with some of the prior mammalian results [17, 37, 38], all 6 PIs displayed inhibitory activities against the _wt_PR. However, besides DRV, only the UIC-94003 and the GRL-0489A showed appreciable suppression activities against the _M7_PR. None of the 6 PIs showed inhibitory activities against the _M10_PR and the _M11_PR that contained high levels of numbers of *PR* multidrug resistance.

Altogether, these data suggested that the _*mdr*_ *PR*-expressing fission yeast cells described here are suitable to be used as a cell-based system to measure the _mdr_PR-specific activities or to test the new PIs against MDR.

## Discussion

In this study, we demonstrated that three clinical isolates of the _mdr_PR (_M7_PR, _M10_PR and _M11_PR), when produced in the fission yeast, maintained their abilities to proteolyze their natural viral substrates, i.e., the MA↓CA (DSQNY↓PIVQ) or the p6 (DSFNF↓PQIT) (Fig. 1B). While they kept their normal enzymatic functions in fission yeast, they also retained their drug resistance. The drug resistant status of these three _mdr_PRs was supported by the observation showing that the IDV treatment suppressed all of the _wt_PR-mediated protein cleavages and cytotoxic activities; whereas it had no suppressive effects on the effects of those _mdr_PRs (Figs. 1, 2, 3). We further showed that the expression of these three _*mdr*_ *PR* genes in the fission yeast prevented the yeast colony formation and cellular growth (Fig. 2A) that ultimately led to cell death (Fig. 2B). Mechanistic analyses suggested that the _mdr_PR-induced cell death were caused by the induction of the ROS production due to the oxidative stress (Fig. 3a) or by the interruption of mitochondrial morphologies (Fig. 3b). Furthermore, the viral PR enzymatic activities appeared to couple with the induction of growth inhibition and cell death, as the IDV and DRV treatments not only prevented the _wt_PR or the _M7_PR-mediated proteolysis (Figs. 1B, 4C), but they also restored cell growth of the fission yeast cells (Figs. 2C, 4B). This intrinsic coupling provided an opportunity for us to use the PR-induced proteolysis and the cell inhibition as the endpoints to measure the PI activities. Indeed, we subsequently showed that the _2nd_PIs, DRV or its five derivatives were able to inhibit the _M7_PR-mediated activities in addition to the _wt_PR (Fig. 4; Additional file 1: Figure S1). Those results were in general agreement with the inhibitory profiles of these compounds in mammalian cells [17, 37, 39]. Note that the three _mdr_PRs described here are naturally occurring PRs that were isolated directly from the HIV-infected patients. Thus one significant finding we reported here is the fact that two of the _mdr_PRs (_M10_PR and _M11_PR) were resistant to all of the existing PI drugs including the _2nd_PI drug, DRV (Table 1, Fig. 4; Additional file 1: Figure S1). DRV was designed and approved by FDA to battle multidrug resistance. This observation certainly underscores the importance of continued searching for new PIs that combat _mdr_PRs [21].

The molecular mechanism of how overexpression of HIV-1 _mdr_PRs cause fission yeast cell death is still not well understood at the moment. However, results of this report on _mdr_PRs and our earlier study on the _wt_PR [32] suggested that _mdr_PR-induced cell death in fission yeast was reminiscent of PR-induced apoptosis in mammalian cells [29, 40]. This was evident by the fact that HIV-1 _mdr_PRs not only triggered the ROS production but also caused mitochondrial changes that were linked to apoptosis [29, 41]. Given the fact that the PIs are able to block _mdr_PR-induced cell death, this cell death must require the PR enzymatic activity. Therefore, it is highly likely that HIV-1 PR-induced cell death in fission yeast is at least in part the result of _mdr_PR enzymatic cleavages of host cellular proteins. Indeed, through a genome-wide search of multicopy suppressor of HIV-1 _wt_PR, we found that overproduction of a fission yeast kinase Hhp2 indeed suppressed _wt_PR-induced cell death in fission yeast [32].

The HIV-1 PR activities have previously been described in both budding yeast and fission yeast [32, 33, 42, 43]. However, only the _wt_PR activities have been shown in fission yeast [32, 33]. The current study was built upon our initial findings on the _wt_PR and further to examine activities of the _mdr_PRs that were isolated from HIV-infected patients. Thus, this is the first report to show multidrug resistance of HIV-1 PR activities in fission yeast. As expected, the _mdr_PRs showed the same enzymatic activities as the _wt_PR, and the overall effects of the _mdr_PRs on fission yeast cellular functions were essentially the same as the _wt_PR. However, all three _mdr_PRs retained their status of drug resistance as they showed in human cells. This observation is significant because it now allows us to use the established fission yeast _mdr_PR-producing cells for the measurement of drug resistance profile of new PIs.

The effects of HIV PRs shown in the fission yeast are somewhat different from that shown in the budding yeast. In budding yeast, HIV-1 PR induces yeast cellular growth arrest that leads to cell lysis. The cell lysis beaks the cell wall and causes the alteration of the plasma membrane, leading to the release of cell contents into the medium [42]. Although HIV-1 PR also prevented cell proliferation and induced cell death in fission yeast (Fig. 2), no cell lysis was seen [33]. This difference between the two yeasts could be due to, at least in part, the relative thick cell wall of fission yeast. Because the fission yeast cells remain intact with the production of HIV-1 PR, the potential advantage of using the fission yeast over the budding yeast is that all of the tests are within cells. Therefore, it is relatively easy to maintain a constant and effective PI drug concentration. The downside of using fission yeast is also because of its thick cell wall. Depending upon the molecular structure of a small molecule, sometimes, much higher PI drug concentrations than that normally used in mammalian cells have to be used to achieve the same inhibitory effect. The actual effective drug concentration varies depending upon the molecular weights and structures of the small molecule. However, this should not be a functional concern for drug testing, because the effects shown by some of the PIs in the fission yeast were in general agreement with the results of mammalian cells [17, 37, 39]. This was further supported by the fact that the IDV effectively inhibited the specific effects of HIV-1 PRs in a dose-dependent manner (Fig. 2A) [32]. Nevertheless, calibration is needed for the comparison of IC50 that is generated from fission yeast with that of mammalian cells. Note that the IC50 can also be calculated in the fission yeast system either based on the % of cellular growth and/or the % of proteolytic cleavages of the substrates induced by HIV-1 PRs [32]. However, there was no need to calculate IC50 here because none of those new PI compounds inhibited the _M10_PR or the _M11_PR.

By using the same fission yeast system, we have previously developed a similar drug testing system for HIV-1 Vpr [31]. That platform was later selected by the NIH’s Molecular Libraries Program. More than 400,000 compounds were successfully screened using the established fission yeast cell-based system. A number of lead compounds were identified as the results. They are currently under evaluation.

There are a number of potential advantages of using fission yeast as a cell-based system to study MDR of HIV-1 PRs. Fission yeast is a simple and unicellular organism with cellular functions that in many ways resemble mammalian cells [44, 45]. For example, fission yeast has been used broadly as a model system to study mammalian cell biology. Knowledge gained from this model organism has contributed significantly to the field of cancer biology [46–48]. Fission yeast cells grow much faster than mammalian cells and are easy to maintain in the laboratory. Because of the simplicity and amenability of the fission yeast, the described _mdr_PR activities in fission yeast could open up the possibilities for us to test new protease inhibiting drugs against those HIV-1 proteases that are already resistant to many of the existing protease inhibitory drugs. Another major advantage of a cell-based assay is that it is able to eliminate those compounds that confer cytotoxicity to yeast cells. Here we have chosen the three _mdr_PRs for the study because they were isolated directly from HIV-infected patients. They represented different levels of naturally occurring MDR to the current antiretroviral regimens (Table 1). By using the low (_M7_PR) and high (_M10_PR or _M11_PR) levels of _mdr_PRs as described here, these new platforms may enable us to evaluate the strength of new PIs against different levels of MDR.

Indeed, in this study, we validated the established fission yeast system by the use of the latest FDA-approved PI drug DRV. The DRV treatment not only inhibited the _wt_PR activities but also reversed the effects of the _M7_PR (Fig. 4). Similar to the DRV effect, all other 5 research grade PIs (UIC-94003, GRL-044-10A, GRL-0249A, GRL-0489A and GRL-0159A) were also able to suppress, with various degrees, the _wt_PR- and the _M7_PR-mediated proteolysis and growth inhibition (Fig. 4B; Additional file 1: Figure S1). In fact, these testing results in the fission yeast were in general agreement with some of the previous mammalian results [17, 37, 39]. For example, similar to the DRV effect, the UIC-94003 also suppressed the _wt_PR-mediated activities in the fission yeast. However, it showed stronger inhibition of the _M7_PR than the DRV in both fission yeast and mammalian cells (Fig. 4B; Additional file 1: Figure S1) [17, 37]. The UIC-94003 was derived from the P2-ligands with incorporation of (R)-(hydroxyethyl)sulfonamide isostere [37]. Interestingly, DRV was actually derived from UIC-94003. It was a product of the combination of the bis-THF ligand and (R)-(hydroxyethyl)sulfonamide isostere with a P2’ sulfonamide functionality [49]. Similarly, a slight better suppression effect of the GRL-0489A than DRV was also observed against the _M7_PR both in the fission yeast and in the mammalian cells (Fig. 4B; Additional file 1: Figure S1) [50]. Different from DRV and UIC-94003, the GRL-0489A was incorporated with a C3-substituted 3-(R)-hydroxyl group [17, 37].

Interestingly, none of these 6 new _2nd_PIs were able to suppress the _M10_PR- and _M11_PR-mediated activities in the fission yeast (Fig. 4B; Additional file 1: Figure S1). However, this finding is not totally surprising because viral multidrug resistance to the existing PIs including the DRV has been previously reported [22–25]. For example, a clinical isolate that contains 20 _mdr_ *PR* gene mutations exhibited extreme resistance to many of the PI drugs [22–24]. An in vitro selection study also showed that high levels of DRV resistance can be artificially selected in the laboratory [25]. What is new here, however, is the fact that the described high level of MDR is not an artificially selected effect but rather an actual clinical situation where those _mdr_PRs were isolated directly from HIV-infected patients.

In summary, we have described activities of three clinically isolated _mdr_PRs in fission yeast. These _mdr_PRs exhibited the same enzymatic activities as the _wt_PR in the fission yeast and in mammalian cells. Moreover, they retained their abilities to confer drug resistance. With the demonstration of their functional relevance and drug resistance in fission yeast, conceivably, we could use these established fission yeast strains for the development of HTS drug screening systems for future discovery of new PIs. The fact that the described _mdr_PRs were isolated directly from HIV-infected patients and none of the six _2nd_PIs including DRV were able to suppress the _M10_PR or _M11_PR, highlights the importance of continued searching for new PIs against MDR. Therefore, our scientific contributions described in this report are two folds: (1) we described, for the first time, a fission yeast cell-based system for HIV-1 _mdr_PRs, and (2) the _mdr_PR we used in our system were novel targets. They were resistant to all of the existing PI drugs including the _2nd_PI drugs such as DRV and they were isolated directly from HIV-infected patients.

## Methods

### Fission yeast strains, plasmid vectors and gene expression

All of the fission yeast stains used in this study are summarized in Table 2. A commonly used wild type fission yeast strain SP223 (*h*-*, ade6*-*216, leu1*-*32, ura4*-*294)* was used to generate all of its derivatives in this study [51]. Standard complete yeast extract with supplement (YES) medium, Edinburgh Minimal Medium (EMM), Pombe Glutamate Medium (PMG) supplemented with adenine, uracil, leucine, thiamine (20 µM), or G418, when necessary, were used for yeast cell growth and plasmid selections.

**Table 2:** Fission yeast strains and plasmids

Strains and plasmids	Genotype and characters	Source or reference
*S. pombe* strains		
SP223	wild type, *h* ^−^ *, ade6*-*216, leu1*-*32, ura4*-*294*	Laboratory collection
Plasmids		
pYZ1N	Fission yeast expression vector with an inducible *nmt1* promoter and a *LEU2* selectable marker	[50]
pYZ2N	Same as pYZ1N but with a *ura4* selectable marker	[50]
pYZ3N	Same as pYZ1N but with a 5′ GFP-tag	[50]
pYZ1N-PR	Wild type HIV-1 *PR* gene cloned in pYZ1N Used to test its effect on cell growth and killing	[32]
pYZ1N-M7	Drug resistant M7 HIV-1 *drPR* gene cloned in pYZ1N Used to test its effect on cell growth and killing	This study
pYZ1N-M10	Drug resistant M10 HIV-1 *drPR* gene cloned in pYZ1N Used to test its effect on cell growth and killing	This study
pYZ1N-M11	Drug resistant M11 HIV-1 *drPR* gene cloned in pYZ1N Used to test its effect on cell growth and killing	This study
pYZ2N-PR	Wild type HIV-1 *PR* gene cloned in pYZ2N Used to test HIV-1 PR proteolytic cleavage	[32]
pYZ2N-M7	Drug resistant M7 HIV-1 *drPR* gene cloned in pYZ2N Used to test HIV-1 PR proteolytic cleavage	This study
pYZ2N-M10	Drug resistant M10 HIV-1 *drPR* gene cloned in pYZ2N Used to test HIV-1 PR proteolytic cleavage	This study
pYZ2N-M11	Drug resistant M11 HIV-1 *drPR* gene cloned in pYZ2N Used to test HIV-1 PR proteolytic cleavage	This study
pYZ3N-GFP-MA-Vpr	GFP is connected to Vpr in pYZ3N by a polylinker containing the PR cleavage MA↓CA site (DSQNY↓PIVQ)	[32]
pYZ3N-p6-MA-Vpr	GFP is connected to Vpr in pYZ3N by a polylinker containing the PR cleavage p6 site (DSFNF↓PQIT)	[32]

Fission yeast expression plasmid vectors pYZ1N and pYZ3N have been previously described [52] and were used in this study for gene expression and proteolytic studies. Both of these two plasmids carry a thiamine repressible no message in thiamine (*nmt1*) promoter where HIV-1 _*mdr*_ *PR* genes were only expressed upon gene induction. Gene expression can be specifically induced or repressed in the absence or presence of 20 µM thiamine [53, 54]. The plasmids pYZ1N and pYZ3N (for GFP-fusion) carry a *LEU2* gene as a selection marker. All yeast plasmid transformation was done by electroporation using a BTX electro cell manipulator (ECM) 600 System protocol 0226 [55]. Anti-HIV PR mouse monoclonal antibody was purchased from Abcore Co. (Ramona, CA; Cat NO. 11-302-C100).

### Isolation of multidrug resistant HIV-1 proteases (_mdr_PR) and protease inhibitors (PIs)

All of the multi-drug resistant HIV-1 protease genes were isolated from normally discarded clinical plasma samples of HIV-1 infected patients who were cared at the University of Maryland Medical Center. This study was thus considered as waived study by the Institutional Review Board because all of the patient’s protected health information has been removed. Genotypic and predicted drug resistant profiles of the isolated HIV-1 mutant proteases are summarized in Table 1. The wild type HIV-1 protease gene was isolated from the commonly used HIV-1 plasmid pNL4-3 and used as a control in this study.

An FDA-approved _1st_PI drug, Indinavir (IDV, Crixivan™), was used primarily to demonstrate drug resistance of the isolated _mdr_PRs. Another FDA-approved _2nd_PI drug, Darunavir (DRV, Prezista™) was used as a positive control because it has been shown to inhibit a wide spectrum of the drug-resistant proteases [49, 56]. Some of the experimental PIs (UIC-94003, GRL-0489A, GRL-0249A, GRL-0159A, and GRL-044-10A) have been tested in mammalian cells (Fig. 4A) [17, 37, 39]. All PI compounds were dissolved in 5% DMSO and stored in −20 °C with the stock concentration of 10 mM. Unless indicated, 200 µM of the PIs were used in each experiment.

### Synthesis of second generation protease inhibitors

Synthesis and evaluation of the experimental PIs, UIC-94003, GRL-0489A, GRL-0249A and GRL-044-10A have been previously described [17, 37, 39]. Experimental details of the syntheses of PIs GRL-0489A, GRL-0249A and GRl-044-10A have been described [17, 37, 39, 57]. Synthesis of PI UIC-94003 carried out using procedure described previously [58]. Synthesis and characterization of PI, GRL-0159A was carried out by following procedure described previously [59]. The details of the synthesis as well as spectral data for compound UIC-94003 and GRL-0159A are available in the Additional file 2.

### Fission yeast assays

To measure cellular growth and gene induction in fission yeast cells, standard culture techniques were used as previously described [26, 60]. Briefly, all fission yeast cells were grown either in minimal EMM or PMG media. Cells carrying plasmids with the *nmt1* promoter were maintained selectively in appropriately supplemented media with 20 μM thiamine to silence the gene expression. For the gene induction, cells were first grown to mid-log growth phase in the presence of 20 μM thiamine. Cells were then washed three times with distilled water and diluted to a concentration of approximately 2 × 10^5^ cells/ml in 5 ml of appropriately supplemented EMM/PMG media with (gene-off) or without (gene-on) thiamine. All of the cells were routinely grown at 30 °C with constant shaking of 250–300 rpm. Cell growth was measured at each time point either by manual counting of the cell numbers or by automated measurement of the optical density (OD_650_) using a spectrophotometer.

A fission yeast colony-forming ability assay was used to investigate the effect of _*mdr*_ *PR* gene expression on fission yeast cell proliferation and viability [27, 35]. Briefly, fission yeast cells were prepared the same way as described above for yeast cell growth. An aliquot of the _*mdr*_ *PR*-on or _*mdr*_ *PR*-off liquid culture was collected at the indicated time points after _*mdr*_ *PR* gene induction and was plated onto thiamine-containing (_*mdr*_ *PR*-off) agar plates. The effect of _mdr_PR on colony-forming ability was evaluated 6 days after plating by comparing the colony sizes between the agar plates with or without the _mdr_PR production. The percentage of cells that formed colonies at each time point was calculated from the number of colonies that grew from the _*mdr*_ *PR*-on cells as a percentage of the number of cells originally plated, which was further calibrated by plating efficiency of the _*mdr*_ *PR*-off cells.

HIV-1 _*mdr*_ *PR*-induced cell death was measured in fission yeast by using a commercial live/dead yeast viability kit (Cat. No. L-7009; Invitrogen, Carlsbad, CA) [27, 32]. Briefly, thiamine was removed from a logarithmic phase cell culture as described above. Cells were then diluted to a concentration of 4 × 10^4^ cells/ml, and re-suspended in PMG minimal medium supplemented with or without thiamine to suppress or induce HIV-1 _*mdr*_ *PR* gene. Cell cultures were grown at 30 °C with constant shaking of 300 rpm, collected at 24 h and resuspended in the GH solution (2% d-(+)-glucose +10 mM Na-HEPES, pH 7.2). A 50 µl aliquot of the FUN-1 solution (80 µM) was added to an equal volume of cell suspension. The suspension was further incubated at 30 °C for 45 min. About 3 µl of the suspension was applied onto a glass slide, covered with a coverslip and sealed with wax. Status of the cell viability was examined by using a Leica DM fluorescent microscopy with 11001v2 long path Chroma filter cube. Typically, actively respiring cells are marked clearly with orange-red fluorescent structures at the maximum wavelength of approximately 590 nm; whereas metabolically inert or dead cells exhibit bright, diffuse, green-yellow fluorescence at the maximum wavelength of approximately 540 nm [27, 32]. FUN1 stained cell images were collected at the excitation wavelength of 470 ± 20 nm with red, green and blue filter set to generate color images by fluorescence merging.

The induction of cellular oxidative stress by _mdr_PR was determined by the production of ROS, which can be detected by an ROS-specific dye, dihydroethidium (DHE, Sigma) that produces red fluorescence in the presence of ROS as described previously [29, 33, 61]. Cells were grown as described above. Twenty-four hours after _*mdr*_ *PR* expression, DHE was added at a concentration of 5 μg/ml and the ROS were detected by fluorescence microscopy.

The mitochondrial morphology of fission yeast was visualized using a vital dye 2-(4-dimethylaminostyryl)-1-methylpyridinium iodide (DASPMI, Sigma) [29, 41]. The _wt_PR and the _*mdr*_ *PR* gene expressions were induced for 24–36 h as described above. Prior to the observation, DASPMI was added to the cells at a concentration of 75 μg/ml. Cells were incubated at 36 °C for 5 min, recovered by centrifugation in a microcentrifuge for 30 s at 500 g, resuspended in 20 μl of YES, and then examined immediately with the fluorescence microscope (L5 filter) at an excitation wavelength of around 470 nm, emission wavelength of 560–570 nm.

### Fluorescence microscopy

A Leica fluorescence microscope DMR4500B equipped with a high performance CCD camera (Hamamatsu) and Open-Lab software (Improvision, Inc., Lexington, MA) was used for all of the imaging analyses. Fission yeast cells were collected onto a regular glass slide and covered with a cover slip. For the observation of green fluorescence, we used a Leica L5 filter with excitation wavelength of 480/40 and emission wavelength of 527/30. For red fluorescence, we used a Leica N2.1 filter with excitation wavelength of 537.5/22.5 and emission wavelength of LP590. To observe green-yellow fluorescence, we used a Leica YFP filter with excitation wavelength of 500/20 and emission wavelength of 535/30.

### Measurements of HIV-1 _mdr_PR activities and substrate specificities in fission yeast

To test whether _mdr_PRs could recognize and cleave the same viral protein recognition sites in fission yeast as the _wt_PR, we developed a “GFP re-localization assay” that allows us to specifically measure proteolytic activities of _mdr_PRs. Briefly, two of the “GFP-MA-Vpr” and the “GFP-p6-Vpr” gene fusion constructs were generated in the fission yeast expression vector pYZ3N [52]. In each of the constructs, GFP and Vpr proteins are connected by a polypeptide linker that contains either a consensus HIV-1 MA↓CA (DSQNY↓PIVQ) or a p6 (DSFNF↓PQIT) PR cleavage sequence [34]. GFP is used for fluorescent detection and it typically disperses throughout fission yeast cells [35, 52]. HIV-1 Vpr is predominantly localized on the nuclear membrane in fission yeast [35, 52] (also see Fig. 1A). Consequently, fusion protein production, without the protease cleavage, appears predominantly as a “ring-like” structure on the nuclear membrane because of Vpr, i.e., the “Vpr pattern” [35, 52]. In contrast, separation of GFP from Vpr due to the _mdr_PR cleavage at the substrate site leads to the “GFP pattern” with uniform distribution throughout cells [35, 52]. To examine the _mdr_PR enzymatic activities, fission yeast cells were prepared as described above and collected 20 h after gene induction.

## Additional files

**Additional file 1: Figure S1.** DRV and its derivatives suppress the _M7_PR but not the _M10_PR or _M11_PR over time. The chemical structures of protease inhibitors, DRV, UIC-94003, GRL-0489A, GRL-0249A, GRL-0159A and GRL-044-10A are shown in (Fig. 4A). All six compounds including DRV are P2 ligands [17]. Effects of the newly synthesized protease inhibitors on _mdr_PR-induced growth arrest were measured against the _wt_PR and _mdr_PRs by using a liquid growth assay and measured by OD_650_ over a time period from 16 to 72 h after the drug treatments and gene inductions. The final drug concentration of 200 µM was used in each of the experiments. DRV was used here as a positive control and no drug treatment was used as a negative control.

**Additional file 2.** Analytical data for protease inhibitors GRL-0159A and UIC-94003.

## Abbreviations

HIV-1: human immunodeficiency virus type 1
PR: protease
PI: protease inhibitor
IDV: indinavir
DRV: darunavir
MDR: multidrug resistance
ARTs: antiretroviral therapies
HTS: high throughput screening
GFP: green fluorescent protein
Vpr: viral protein R
ROS: oxidative stress species
