# Supplementary figures and images for: A fission yeast cell-based system for multidrug resistant HIV-1 proteases
Source: Cell Biosci. 2017 Jan 11;7:5. doi: 10.1186/s13578-016-0131-5 (PMC5225522; doi:10.1186/s13578-016-0131-5)

## Slide 1
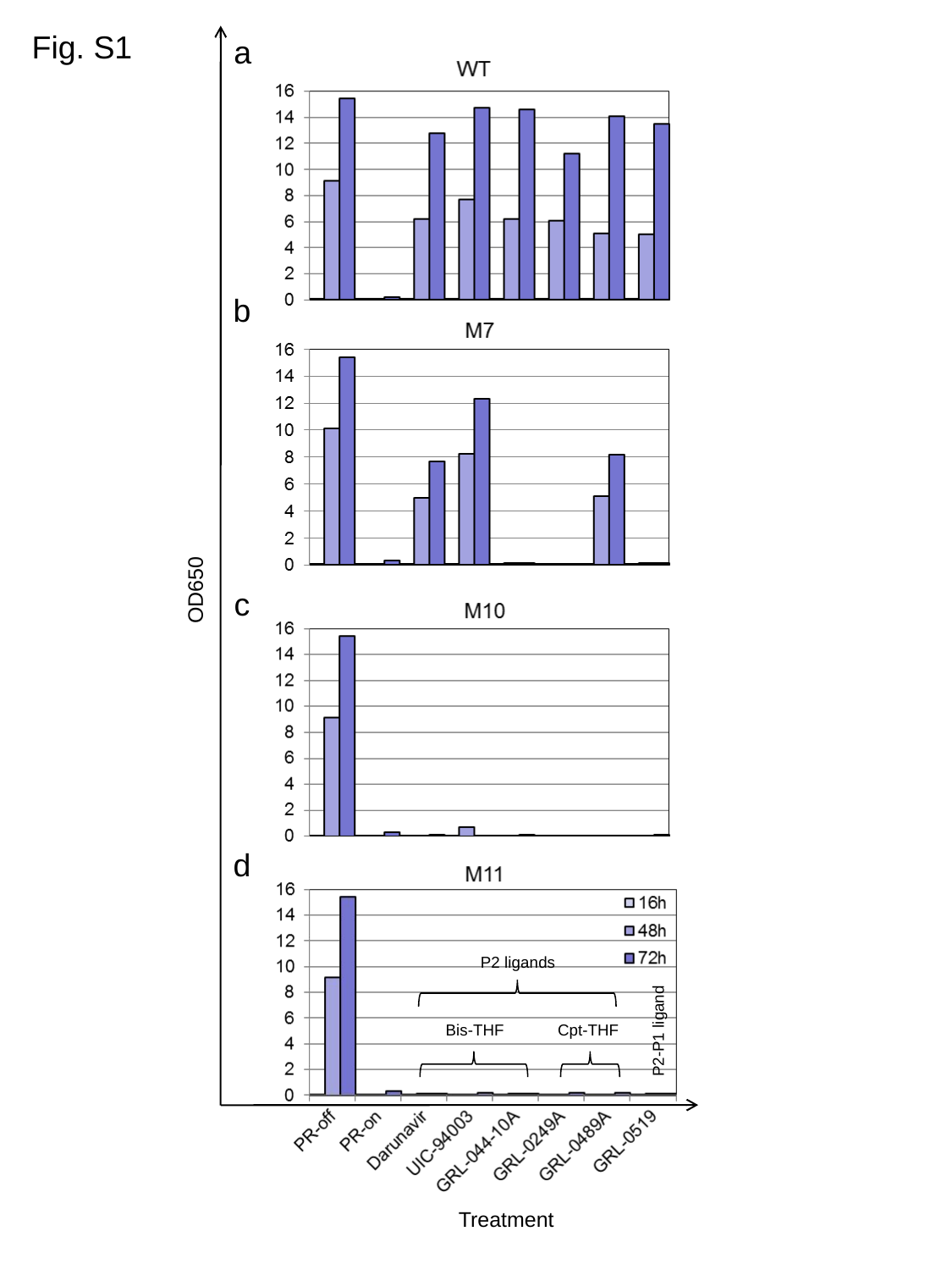

Fig. S1
a
b
OD650
c
d
P2 ligands
Bis-THF
Cpt-THF
P2-P1 ligand
Treatment

Supplement: Supplementary file 1 — Additional file 1: Figure S1. DRV and its derivatives suppress the M7PR but not the M10PR or M11PR over time. The chemical structures of protease inhibitors, DRV, UIC-94003, GRL-0489A, GRL-0249A, GRL-0159A and GRL-044-10A are shown in (Fig. 4A). All six compounds including DRV are P2 ligands [17]. Effects of the newly synthesized protease inhibitors on mdrPR-induced growth arrest were measured against the wtPR and mdrPRs by using a liquid growth assay and measured by OD650 over a time period from 16 to 72 h after the drug treatments and gene inductions. The final drug concentration of 200 µM was used in each of the experiments. DRV was used here as a positive control and no drug treatment was used as a negative control. [file 13578_2016_131_MOESM1_ESM.ppt]
