# Supplementary material for: A fission yeast cell-based system for multidrug resistant HIV-1 proteases
Source: Cell Biosci. 2017 Jan 11;7:5. doi: 10.1186/s13578-016-0131-5 (PMC5225522; doi:10.1186/s13578-016-0131-5)
Supplement: Supplementary file 2 — Additional file 2. Analytical data for protease inhibitors GRL-0159A and UIC-94003. [file 13578_2016_131_MOESM2_ESM.docx]

**Supporting Information Text 1.** Analytical Data for Protease Inhibitors GRL-0159A and UIC-94003

**GRL-0159A:** *3-((2R,4R)-4-(benzyloxy)-2-(methoxymethyl)pyrrolidine-1-carbonyl)-N-((2S,3R)-3-hydroxy-4-((N-isobutyl-4-methoxyphenyl)sulfonamido)-1-phenylbutan-2-yl)benzamide (****159A****)*. ^1^H NMR (400 MHz, CDCl_3_) δ 7.71 (s, 1H), 7.69-7.64 (m, 3H), 7.58 (d, *J* = 7.6 Hz, 1H), 7.43 (t, *J* = 7.7 Hz, 1H), 7.29-7.28 (m, 7H), 7.26 (s, 2H), 7.20 (dd, *J* = 5.1, 3.2 Hz, 1H) 6.96 (d, *J* = 8.8. Hz, 2H), 6.49 (d, 8.2 Hz, 1H), 4.53 (d, *J* = 11.1 Hz, 2H), 4.41 (dd, *J* = 13.1, 8.3, 2H), 4.18 (s, 1H), 4.00 (d, *J* = 17.7 Hz, 2H), 3.86 (s, 3H), 3.82 (d, *J* = 19.5 Hz, 2H), 3.62-3.50 (m, 1H), 3.42 (s, 3H), 3.14-3.07 (m, 4H), 2.90-2.86 (m, 2H), 2.24 (s, 2H), 1.89-1.85 (m, 1H), 1.72 (brs, 1H), 0.87 (t, *J* = 6.4 Hz, 6H); ^13^C NMR (100 MHz, CDCl_3_) δ 167.1, 163.0, 137.6, 137.2, 134.3, 130.3, 129.7, 129.3, 128.6, 128.4, 127.8, 127.6, 126.6, 125.6, 114.3, 72.7, 72.3, 71.4, 58.8, 55.5, 55.0, 54.5, 53.5, 35.0, 32.4, 27.2, 20.0, 19.9; LRMS-ESI (*m*/*z*) 780.8 [M + Na]^+^.

**UIC-94003***: (3R,3aS,6aR)-hexahydrofuro[2,3-b]furan-3-yl ((2S,3R)-3-hydroxy-4-((N-isobutyl-4-methoxyphenyl)sulfonamido)-1-phenylbutan-2-yl)carbamate.* ^1^H NMR (400 MHz, CDCl_3_) δ 7.71 (d, *J* = 8.8. Hz, 2H), 7.28-7.26 (m, 2H), 7.23-7.21 (m, 3H), 6.99 (d, *J* = 8.8. Hz, 2H), 5.64 (d, *J* = 5.2 Hz, 1H), 5.02 (q, *J* = 6.7 Hz, 1H), 4.94 (d, *J* = 8.8 Hz, 1H), 3.95 (dd, *J* = 9.7, 6.3 Hz, 1H), 3.88 (s, 3H), 3.85-3.83 (m, 3H), 3.75 – 3.63 (m, 3H), 3.17 (dd, *J* = 15.1, 8.5 Hz, 1H), 3.08 (dd, *J* = 14.2, 4.3 Hz, 1H), 3.00 (dd, *J* = 5.5, 2.9 Hz, 1H), 2.98 – 2.94 (m, 1H), 2.90 (td, *J* = 9.6, 8.5, 4.9 Hz, 1H), 2.81 (d, *J* = 6.5 Hz, 1H), 2.79 – 2.76 (m, 1H), 1.83 (dt, *J* = 14.0, 7.0 Hz, 1H), 1.47 (dd, *J* = 12.6, 5.6 Hz, 1H), 0.91 (dd, *J* = 20.0, 6.6 Hz, 6H); ^13^C NMR (100 MHz, CDCl_3_) δ 163.0, 155.4, 137.5, 129.6, 129.4, 129.2, 128.5, 126.5, 114.3, 109.2, 73.4, 72.7, 70.7, 69.5, 58.8, 55.6, 55.0, 53.7, 45.2, 35.6, 27.2, 25.7, 20.1, 19.8; LRMS-ESI (*m*/*z*) [M + Na]^+^.
